# Cytochrome b5 protects photoreceptors from light stress-induced lipid peroxidation and retinal degeneration

**DOI:** 10.1038/s41514-017-0019-6

**Published:** 2017-12-04

**Authors:** Xinping Chen, Hana Hall, Jeffrey P. Simpson, Walter D. Leon-Salas, Donald F. Ready, Vikki M. Weake

**Affiliations:** 1 Department of Biochemistry, Purdue University, West Lafayette, IN 47907 USA; 2 Purdue Polytechnic Institute, Purdue University, West Lafayette, IN 47907 USA; 3 Department of Biological Sciences, Purdue University, West Lafayette, IN 47907 USA; 4 Purdue University Center for Cancer Research, Purdue University, West Lafayette, IN 47907 USA; 5 Present Address: University of Notre Dame, Notre Dame, IN 46556 USA

## Abstract

Lipid peroxides are generated by oxidative stress in cells, and contribute to ageing and neurodegenerative disease. The eye is at special risk for lipid peroxidation because photoreceptors possess amplified sensory membranes rich in peroxidation-susceptible polyunsaturated fatty acids. Light-induced lipid peroxidation in the retina contributes to retinal degeneration, and lipid peroxidation has been implicated in the progression of age-associated ocular diseases such as age-related macular degeneration (AMD). Here, we show that exposing *Drosophila melanogaster* to strong blue light induces oxidative stress including lipid peroxidation that results in retinal degeneration. Surprisingly, very young flies are resilient to this acute light stress, suggesting they possess endogenous neuroprotective mechanisms. While lipophilic antioxidants partially suppressed blue light-induced retinal degeneration in older flies, we find that overexpression of cytochrome b5 (Cyt-b5) completely suppressed both blue light-induced lipid peroxidation and retinal degeneration. Our data identify Cyt-b5 as a neuroprotective factor that targets light-induced oxidative damage, particularly lipid peroxidation. Cyt-b5 may function via supporting antioxidant recycling, thereby providing a strategy to prevent oxidative stress in ageing photoreceptors that would be synergistic with dietary antioxidant supplementation.

## Neuroscience: Vision is stressful for old flies

Paradoxically, light is essential for vision, yet it also induces stress that damages the sensitive cells in the eye. Vikki Weake and her team at Purdue University examined how exposure to blue light causes damage to the retina in fruit flies. Blue light causes death of photoreceptors, the light-sensing neurons. Surprisingly, very young flies are resistant to blue light. Increasing levels of a single protein, Cytochrome-b5, mimicked youthful resilience in older flies. Cytochrome-b5 is central to an ancient cellular defense system that protects membranes from oxidative damage. With expansive sensory membranes containing specialized lipids, photoreceptors are especially sensitive to membrane lipid peroxidation, an emerging final common pathway for cell death in aging and disease. Research into preventing lipid peroxidation might help to develop therapies for age-related diseases such as age-related macular degeneration.

## Introduction

During ageing, weakened antioxidant defenses allow accumulation of toxic reactive oxygen species (ROS) that contribute to ageing, and to multiple diseases, including cancer, neurodegeneration and age-related macular degeneration (AMD).^1–5^ Once initiated by any of several pathways, lipid peroxidation, oxidative damage of membrane lipids, spreads aggressively in a self-propagating chain reaction, amplifying oxidative damage.^6^ Lipid peroxides adversely alter membrane structure and function and generate highly reactive toxic secondary products that react with proteins and DNA, compromising normal activity.^6^ The retina is uniquely at risk for lipid peroxidation because of its high concentration of peroxidation-sensitive polyunsaturated fatty acids, and energy-intensive, oxygen-rich environment.^4^ Multiple pathways, including photodynamic generation of ROS by rhodopsin and associated metabolites, translate light into retinal oxidative stress;^7^ constant light causing photoreceptor degeneration in rat retina generates lipid peroxides in photosensory outer segment membranes.^8^ Antioxidants that terminate radical propagation, such as vitamin E, or glutathione peroxidases that reduce lipid peroxides form the major defense mechanisms against lipid peroxidation^6^ and antioxidant supplements are the standard of care to slow AMD progression.^9^ However, antioxidant supplements do not halt AMD progression,^9^ and antioxidant therapy has not shown positive results in intervention trials for other neurodegenerative diseases involving oxidative stress.^5,10^ Identifying factors that enhance the ability of neurons to cope with oxidative stress could provide therapeutic avenues for age-related neurodegenerative diseases, including AMD.

Here, we describe an acute phototoxicity model in the fruitfly, *Drosophila melanogaster*, in which blue light exposure induces phototransduction-dependent oxidative stress, lipid peroxidation and retinal degeneration. We identify Cyt-b5 as a neuroprotective factor that prevents retinal degeneration by suppressing light-stress-induced lipid peroxidation. Cyt-b5 partners with Cyt-b5 reductase to form the plasma membrane redox system, an ancient front line defense against lipid peroxidation.^2^ Thus, potentiation of the plasma membrane redox system via targeted Cyt-b5 expression offers a strategy, potentially synergistic with dietary antioxidants, to delay the onset or progression of age-related neurodegenerative diseases involving lipid peroxidation.

## Results

**Fig. 1:**
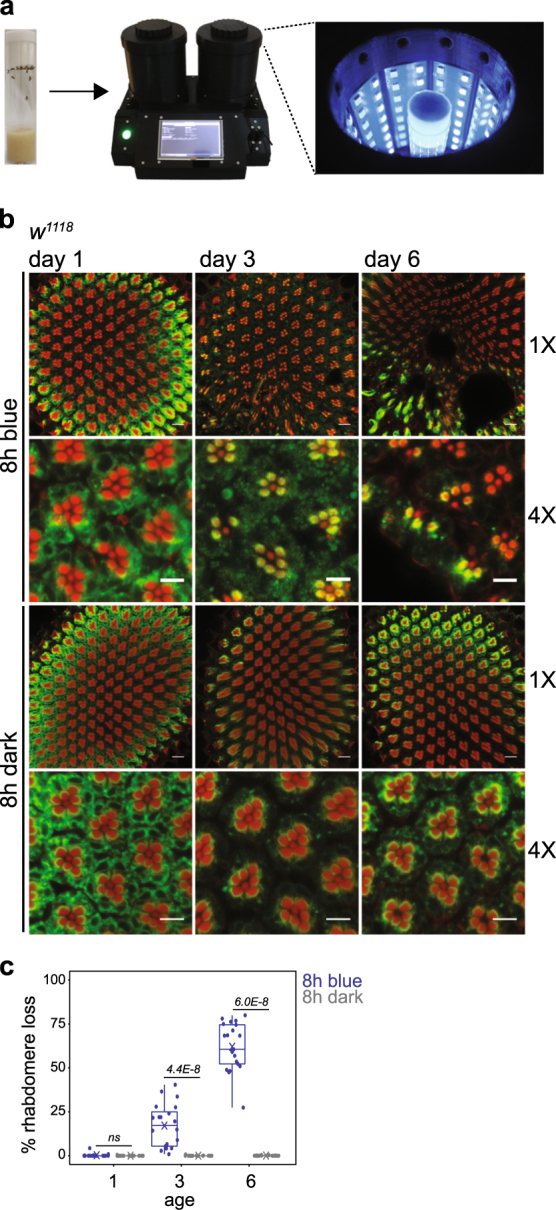
Flies show an increase in blue light-induced retinal degeneration between 1 and 6 days post-eclosion. **a** A custom designed optical stimulator was used to expose flies to blue light at 2 mW/cm^2^. Male flies were raised in 12 h/12 h light/dark conditions for 1–6 days prior to blue light exposure or dark control. **b** Confocal images of adult retinas stained with phalloidin (red) and 4C5 (Rh1, green) from male white-eyed (*w* ^*1118*^) flies 1, 3 and 6 days post-eclosion exposed to 8 h blue light or dark (control). Retinas were dissected and immunostained following 12 days dark incubation post-treatment to assess rhabdomere loss. Scale bars: 1×, 10 μm; 4×, 5 μm. Unless otherwise stated, these details are constant for all confocal images presented in subsequent figures. **c** Box plots showing rhabdomere loss quantified using confocal images. Means are shown by crosses. Points are overlayed for individual animals (single eye/animal) representing 4 independent light treatments with 5 flies per treatment. The distribution for each blue light-treated group was compared with the dark control for the same age using Kruskal–Wallis test followed by pairwise Wilcoxon Rank Sum test with Benjamini and Hochberg correction, and the false discovery rate (FDR) for each comparison is shown. ns not significant. Unless otherwise stated, these details are constant for all box plots presented in subsequent figures

**Fig. 2:**
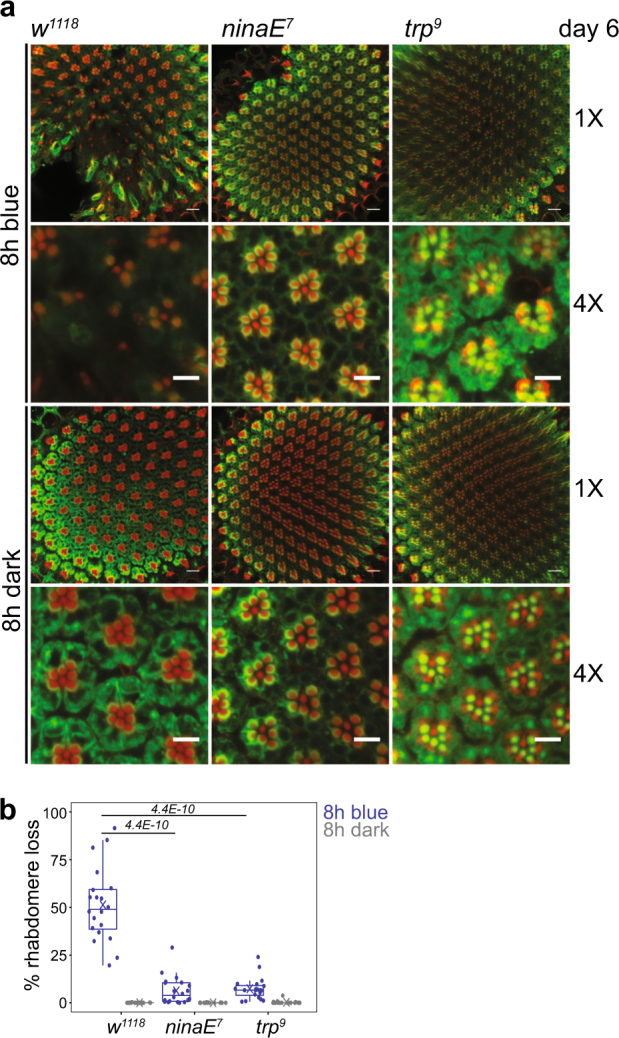
Blue light-induced retinal degeneration requires an intact phototransduction pathway and Ca^2+^ influx. **a** Confocal images of adult retinas from *w* ^*1118*^ *, ninaE* ^*7*^ and trp^9^ flies exposed to 8 h blue light or dark control at 6 days post-eclosion. Flies were maintained in the dark prior to blue light exposure to prevent light-induced degeneration in the mutant backgrounds. **b** Box plots showing rhabdomere loss quantified using the confocal images. FDR, pairwise Wilcoxon Rank Sum Test between wild type (*w* ^*1118*^) and mutant genotypes under blue light treatment (*n* = 4 light treatments; 5 animals/experiment)

To establish conditions that reliably elicited photoreceptor degeneration in response to light stress, we exposed flies to regimes of varying light intensity and duration at different ages and examined their eyes post-exposure using confocal and electron microscopy (Fig. 1a). As flies lacking normal eye screening pigment are sensitized to light damage,^11^ we used white-eyed (*w* ^*1118*^) flies. We found eight hours of strong blue light (*λ* = 465 nm) caused loss of the photosensory membrane organelle (rhabdomere) in fewer than 1% of photoreceptors in 1-day-old flies (12–19 h post-eclosion), but 17% and 62% by 3 and 6 days of age respectively (Fig. 1b, c). We previously showed that exposure to similar intensities of red light did not induce retinal degeneration in 6-day-old *w* ^*1118*^ flies,^12^ indicating that blue light specifically induces retinal degeneration. Strong blue light photoconverts the bulk of the light-sensitive G-protein-coupled receptor Rhodopsin 1 (Rh1) in the outer photoreceptors to its active form, metarhodopsin (M).^13^ In the absence of orange light (*λ* = 580 nm), which reverts M back to Rh1, prolonged phototransduction occurs.^13,14^ To test if phototransduction was required for degeneration, we examined flies carrying mutations that strongly reduce Rh1 levels (*ninaE* ^*7*^). We observed that *ninaE* ^*7*^ mutations suppressed rhabdomere loss in 6-day-old flies exposed to blue light (Fig. 2a, b), indicating that phototransduction is necessary for blue light-induced retinal degeneration. In flies, light triggers the phototransduction cascade in which signaling initiated via Rh1 culminates in opening of Trp calcium (Ca^2+^) channels and influx of Ca^2+^ into photoreceptor neurons.^13^ To test if Ca^2+^ influx was required for blue light-induced retinal degeneration, we examined *trp* ^*9*^ flies. Similar to *ninaE* ^*7*^, *trp* ^*9*^ mutations suppressed rhabdomere loss (Fig. 2a, b), indicating that phototransduction-activated Ca^2+^ influx and cytosolic Ca^2+^ overload is the proximal cause of degeneration in 6-day-old flies. This observation is consistent with findings that unregulated Ca^2+^ influx via constitutively active mutant *Trp* ^*365*^ channels causes photoreceptor degeneration^15^ and overexpression of CalX, which increases Ca^2+^ extrusion, suppresses this degeneration.^16^ One-day-old flies are thus resilient to blue light stress-induced Ca^2+^ cytotoxicity.

**Fig. 3:**
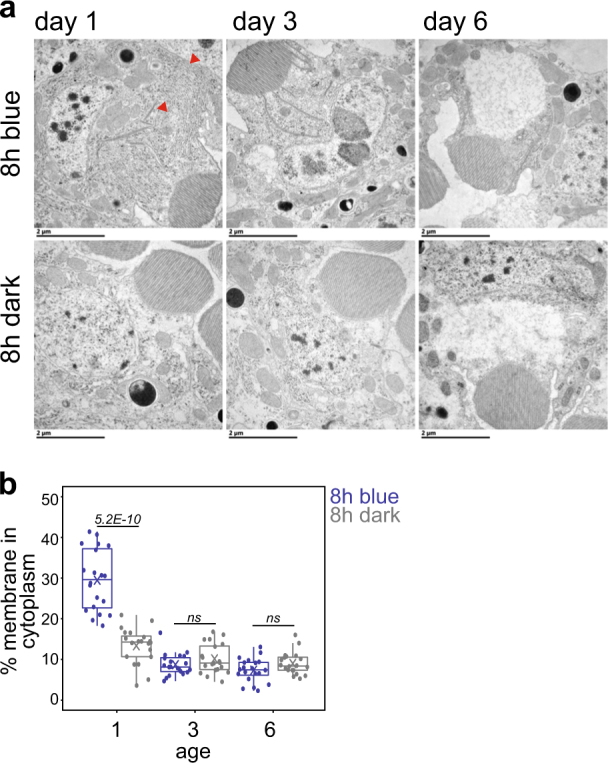
Blue light induces endoplasmic reticulum expansion in day 1 flies. **a** Transmission electron microscopy of cross sections of ommatidia from adult *w* ^*1118*^ flies of the indicated ages exposed to 8 h blue light or dark (control). Sections containing nuclei for a single R1–R6 cell are shown. Red arrowheads indicate expanded intracellular membranes. Scale bars: 2 μm. **b** The percentage of cytoplasm containing intracellular membranes was quantified using multiple electron microscopy images representing multiple ommatidia (planes) for each eye (*n* = 4 light treatments; 5 animals/treatment; 1 ommatidium/animal). FDR, pairwise Wilcoxon Rank Sum Test

**Fig. 4:**
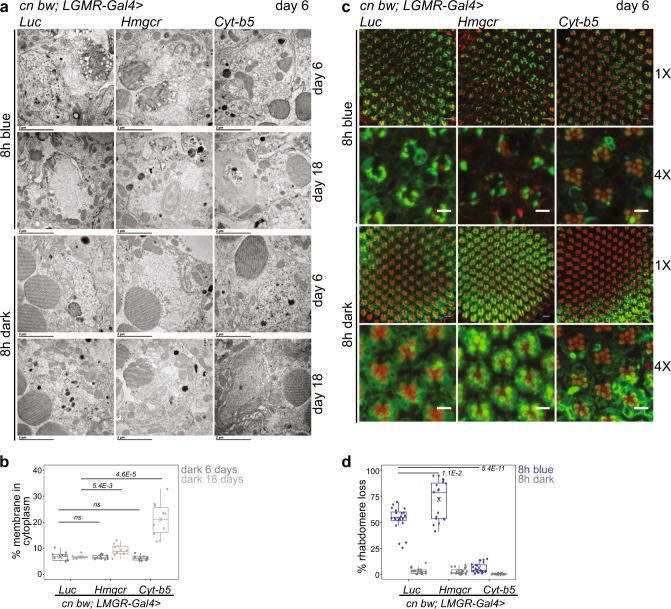
Overexpression of Cyt-b5 suppresses blue light-induced retinal degeneration. **a** Transmission electron microscopy of cross sections of ommatidia from adult male flies expressing single copy transgenes for *Luciferase (Luc)*, *Hmgcr,* or *Cyt-b5* driven by *LGMR-Gal4*. All *mini-white* marked transgenes were crossed to *cn bw* to deplete eye pigments. Flies were exposed to blue light exposure or dark control at 6 days post-eclosion and retinas were either dissected immediately (day 6) or after a further 12 days of dark incubation (day 18). Sections containing nuclei for a single R1–R6 cell are shown. Scale bars: 2 μm. **b** The percentage of cytoplasm containing intracellular membranes was quantified for dark treated animals at 6 days or 18 days post-eclosion using the electron microscopy images. FDR, pairwise Wilcoxon Rank Sum Test between Luciferase and Hmgcr or Cyt-b5 for the same age (*n* = 3 animals; 3–4 ommatidia/animal). **c** Confocal images of adult retinas from male flies of the indicated genotypes exposed to 8 h blue light or dark control. **d** Box plots showing rhabdomere loss quantified using the confocal images. FDR, pairwise Wilcoxon Rank Sum Test between Luciferase and Hmgcr or Cyt-b5 under blue light treatment (*n* = 4 light treatments; 5 animals/experiment). ns not significant

The endoplasmic reticulum (ER) regulates cellular Ca^2+^ homeostasis and cytosolic Ca^2+^ overload causes ER stress.^17–19^ Fly photoreceptors lacking the Ca^2+^-buffering protein Calphotin show ER stress, enhanced autophagy, and strong, light-dependent ER amplification.^20^ We observed increased ER-like membranes in photoreceptors from 1-day-old flies, but not older flies, exposed to blue light (Fig. 3a, b). Since the ER expansion in 1-day-old flies exposed to blue light correlated with their resistance to blue light-induced retinal degeneration, we sought to test whether this increased ER biogenesis was neuroprotective. To test this hypothesis, we used the eye-specific *LGMR-Gal4* driver to overexpress Cytochrome b5 (Cyt-b5, previously identified as *dappled* ^21^) or HMG Coenzyme A reductase (Hmgcr^22^), which are both reported to induce ER biogenesis.^23,24^ We did not observed expansion of ER-like intracellular membranes by day 6 post-eclosion in flies overexpressing Cyt-b5 or Hmgcr in the eye (Fig. 4a, b). However, by 18 days post-eclosion, overexpression of both Cyt-b5 and Hmgcr significantly increased ER-like membranes in photoreceptors relative to the Luciferase control (Fig. 4a, b). Strikingly, in 6-day-old flies that did not show expanded ER, overexpression of Cyt-b5, but not Hmgcr or Luciferase, strongly suppressed blue light-induced retinal degeneration (Fig. 4c, d). To confirm that Cyt-b5 was overexpressed by day 6, we examined *Cyt-b5* transcript levels in eyes from Cyt-b5 overexpressing flies at day 6. Notably, *Cyt-b5* transcript levels were ~18-fold higher than the Luciferase control at 6 days post-eclosion (Supplementary Fig. 1). Further, the suppression of blue light-induced retinal degeneration was not due to position effects of the *UAS-Cytb5* transgene because in the absence of the *LGMR-Gal4* driver, *UAS-Cytb5* flies showed blue light-induced retinal degeneration (Supplementary Fig. 2). If the neuroprotective mechanism from blue light stress involved ER expansion, then we would expect that both Cyt-b5 and Hmgcr would be neuroprotective. However, the modest but significant membrane expansion in Hmgcr-overexpressing flies enhanced, rather than suppressed, blue light-induced retinal degeneration. Further, we did not observe expanded ER by day 6 when Cyt-b5 exerted its protective effect. Thus, ER stress-induced membrane biogenesis alone is not sufficient to protect photoreceptors from blue light-induced retinal degeneration. Rather, our data suggest that Cyt-b5 protects photoreceptors from degeneration through another mechanism.

**Fig. 5:**
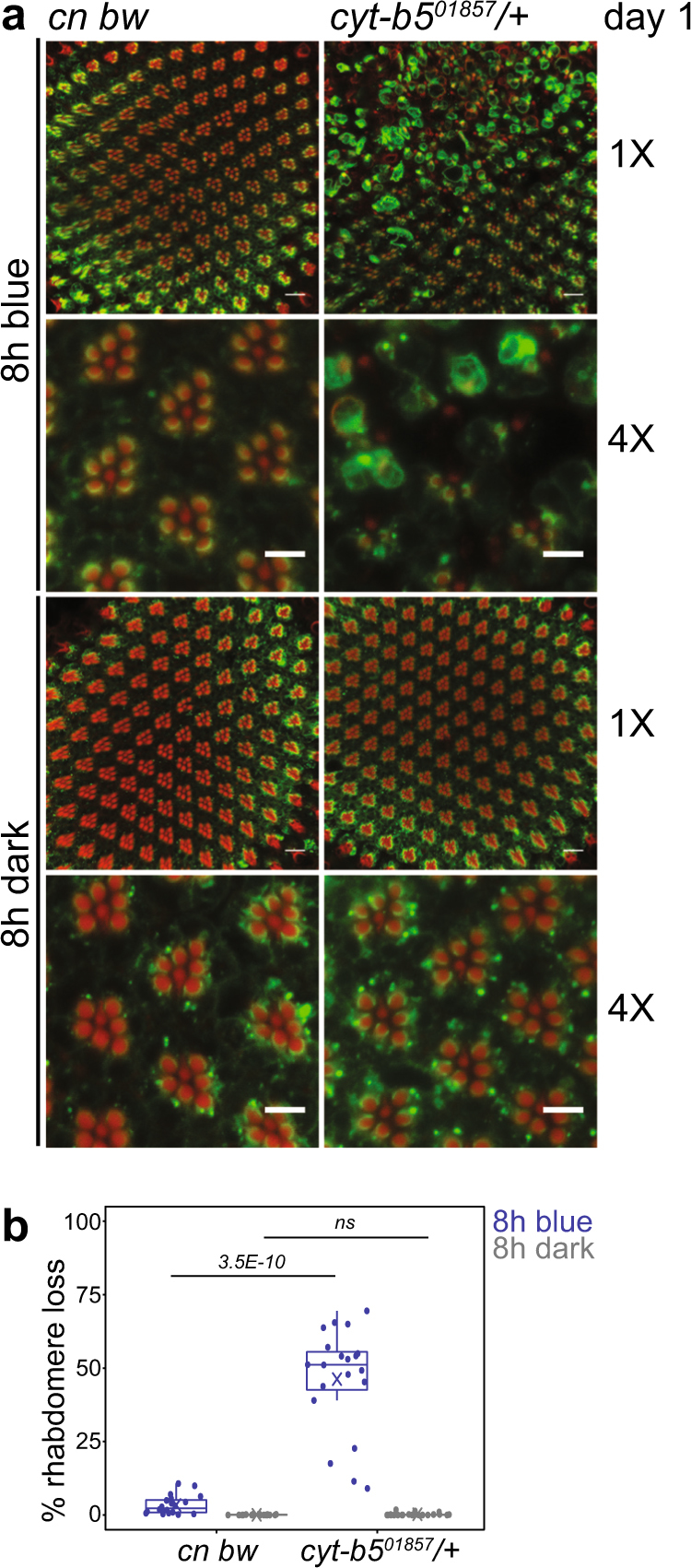
Flies with reduced Cyt-b5 show enhanced blue light-induced retinal degeneration. **a** Confocal images of adult retinas from day 1 flies heterozygous for *cytb5* ^*01857*^ in the *cn bw* background vs. *cn bw* flies (control) exposed to 8 h blue light or dark control. **b** Box plots showing rhabdomere loss quantified using the confocal images. FDR, pairwise Wilcoxon Rank Sum Test between different genotypes under the same light treatment (*n* = 4 light treatments; 5 animals/experiment). ns not significant

Cyt-b5 is an ER and plasma membrane-associated heme-containing protein that transfers electrons in multiple biochemical pathways including fatty acid desaturation, cytochrome P450-reactions, and sterol metabolism.^25^ Cyt-b5 is required for synthesis of fatty acids including palmitoleic acid (16:1Δ), which has been shown to protect the mouse retina from light-induced damage.^26^ However, overexpression of Cyt-b5 did not alter the ratio of polyunsaturated fatty acids, or increase relative levels of palmitoleic acid, in the eye (Supplementary Fig. 3). Thus, Cyt-b5 is unlikely to suppress blue light stress-induced retinal degeneration via altering polyunsaturated fatty acid levels. Therefore, we sought to identify alternative mechanisms through which Cyt-b5 could suppress blue light-induced retinal degeneration. Cyt-b5 is the rate-limiting electron-donor for the NADH-dependent reduction of membrane resident antioxidant coenzyme Q, which quenches lipid peroxidation and protects cells from oxidative stress.^27,28^ Because their photosensory membranes are rich in susceptible polyunsaturated fatty acids, photoreceptors are at special risk for damage by lipid peroxidation, a ROS-induced peroxyl radical chain reaction that propagates across cell membranes, compromising membrane organization and cellular integrity. We hypothesized that Cyt-b5 overexpression promotes coupling of Cyt-b5 and its reductase, enhancing plasma membrane antioxidant defenses. Based on this hypothesis, we predicted that decreased Cyt-b5 levels would diminish resilience to blue light-induced oxidative stress in very young flies. To test this, we examined *cyt-b5* ^*01857*^ heterozygotes carrying one copy of a strong hypomorphic allele of *Cyt-b5* and found that these flies showed strongly enhanced blue light-induced rhabdomere loss in day 1 flies (Fig. 5a, b). Despite this increase in sensitivity to blue light in flies with decreased *Cyt-b5* levels, we did not observe differences in *Cyt-b5* transcript levels between day 1 and 6 (Supplementary Fig. 1). We were unable to examine Cyt-b5 protein levels due to lack of an antibody that recognizes *Drosophila* Cyt-b5.

**Fig. 6:**
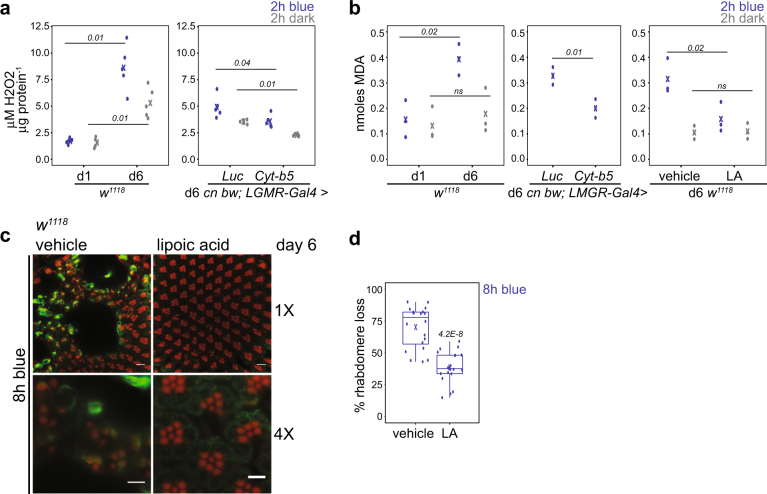
Reducing lipid peroxidation suppresses blue light-induced retinal degeneration. **a** H_2_O_2_ levels were measured in retinas from flies of the indicated ages and genotypes exposed to 2 h blue light or dark control (d1, day 1; d6, day 6). H_2_O_2_ concentrations were determined relative to total amount of protein from 10 eyes, and data are plotted as individual points. FDR, pairwise Wilcoxon Rank Sum Test between different genotypes or ages under the same light treatment (*n* = 5 light treatments). **b** Malondialdehyde (MDA) levels were measured in retinas from flies of the indicated ages, genotypes, or lipoic acid (LA) treatment, and data are plotted as individual points. *p*-values, ANOVA followed by Tukey-HSD between different ages, genotypes or treatment under the same light exposure (*n = 3 light treatments*). ns not significant. **c** Confocal images of adult retinas from *w* ^*1118*^ flies grown on food supplemented with lipoic acid or vehicle (ethanol) for 6 days post-eclosion prior to 8 h blue light exposure. **d** Box plots showing rhabdomere loss quantified using the confocal images. *p*-value, Student’s *t* test (*n *= 4 light treatments; 5 animals/experiment)

If overexpression of Cyt-b5 promotes coupling of Cyt-b5 and its reductase, then we would expect decreased levels of oxidative stress in flies overexpressing Cyt-b5. Previous studies have shown that overexpression of Cyt-b5 reductase extends lifespan in yeast,^29^ flies and mice,^30^ and reduces levels of H_2_O_2_ and lipid peroxidation in transgenic mouse liver.^30^ To test if overexpression of Cyt-b5 reduced oxidative stress, we measured H_2_O_2_ levels in eyes. H_2_O_2_ levels increased in the retina of day 6 flies following blue light treatment, indicating that blue light exposure results in ROS generation (Fig. 6a). The blue light-induced increase in H_2_O_2_ levels was not observed in day 1 flies, which also showed lower basal (dark) levels of H_2_O_2_ as compared with day 6 flies (Fig. 6a). Overexpression of Cyt-b5-reduced H_2_O_2_ levels in the retina of both dark and blue-exposed day 6 flies relative to the Luciferase control (Fig. 6a). Thus, blue light increases ROS levels in the eye, which are suppressed by overexpression of Cyt-b5.

Next, we asked if light-activated Ca^2+^ influx was necessary for the increase in H_2_O_2_ levels following blue light exposure. To do this, we examined H_2_O_2_ levels upon blue light exposure in the *trp* ^*9*^ mutant, which lacks the major Ca^2+^ channel required for influx. The increase in H_2_O_2_ levels upon blue light exposure was suppressed in the *trp* ^*9*^ mutant, indicating that light-induced Ca^2+^ influx is necessary for ROS generation (Supplementary Fig. 4). Since *trp* ^*9*^ mutations also suppress blue light-induced retinal degeneration, these data further support Ca^2+^ influx-driven increases in ROS levels as the proximal cause of retinal degeneration. Based on this model, one potential mechanism for Cyt-b5’s neuroprotective action could be suppression of the prolonged Ca^2+^ influx resulting from blue light exposure. However, overexpression of Cyt-b5 does not prevent light-induced Ca^2+^ influx because flies that overexpress Cyt-b5 showed normal electroretinograms (ERGs) with a clear prolonged depolarizing afterpotential indicative of sustained photoreceptor Ca^2+^ influx (Supplementary Fig. 5). Thus, Cyt-b5 overexpression suppresses blue light-induced oxidative stress without affecting phototransduction or light-induced Ca^2+^ influx. These data are consistent with a neuroprotective mechanism in which Cyt-b5 reduces ROS levels via enhancing activity of Cyt-b5-dependent enzymes such as Cyt-b5 reductase.

It appears unlikely that H_2_O_2_ is the critical ROS target through which Cyt-b5 exerts its neuroprotective activity, since overexpression of Superoxide dismutase 1 (Sod1), which destroys superoxide radicals, decreased blue light-induced H_2_O_2_ levels but did not suppress blue light-induced retinal degeneration (Supplementary Fig. 6). Cyt-b5 is known to enhance NADH-dependent reduction of membrane resident antioxidant coenzyme Q, which directly quenches lipid peroxyl radicals and also regenerates vitamin E, terminating the peroxidation chain reaction.^27,28^ Malondialdehyde (MDA) is a toxic secondary product of lipid peroxidation and marker of membrane oxidative damage; thus, we asked if blue light stress produced MDA and if Cyt-b5 overexpression decreased its production. We measured MDA levels in retinas from day 1 and day 6 flies exposed to blue light and found that blue light indeed increases levels of MDA in day 6 flies, but not in day 1 flies (Fig. 6b). Similar to our observations, mouse models of light-induced damage show extensive rod outer segment lipid peroxidation.^31^ Notably, overexpression of Cyt-b5 suppressed the blue light-induced production of MDA; indeed, the levels of MDA in Cyt-b5 overexpressing day 6 flies resembled those of day 1 flies (Fig. 6b). In view of Cyt-b5’s membrane association, we hypothesized that dietary supplementation with the lipid-associated antioxidant lipoic acid^32^ would protect flies from blue light-induced retinal degeneration. Lipoic acid supplementation suppressed blue light-induced MDA accumulation in day 6 flies (Fig. 6b) and also suppressed blue light-induced retinal degeneration (Fig. 6c, d). Flies supplemented with another antioxidant, vitamin E, which forms part of the current antioxidant therapy used for AMD patients,^9^ also showed lower blue light-induced retinal degeneration than vehicle controls, although to a lesser extent than that observed with lipoic acid (Supplementary Fig. 6). However, neither lipoic acid nor vitamin E treatment were as effective as overexpression of Cyt-b5 at suppressing blue light-induced retinal degeneration. These data show that Cyt-b5 is more protective than traditional antioxidants from an acute phototoxicity model involving blue light stress-induced retinal degeneration in *Drosophila*.

## Discussion

Although lipid peroxidation is a normal component of cell signaling, its dysregulation causes damage that contributes to ageing and degenerative disease.^6^ Oxidized plasma membrane lipids are a hallmark of senescent cells^33^ and oxidative stress contributes to neurodegeneration, and photoreceptor loss in AMD and upon blue light damage.^34,35^ Once initiated, lipid peroxidation spreads in a self-propagating chain reaction, amplifying membrane damage and generating secondary reactive compounds that crosslink DNA and proteins.^6^ The major defense mechanisms against lipid peroxidation are the membrane-targeted chain-breaking antioxidants, reduced coenzyme Q and vitamin E, and glutathione peroxidases that enzymatically reduce lipid peroxides.^6^ Reduced coenzyme Q and, in turn, vitamin E are regenerated in the membrane where coenzyme Q reductase partners with Cyt-b5 to transfer reducing power from cytosolic NADH.^36,37^ Here we show that Cyt-b5 overexpression improves resiliency to acute light stress in *Drosophila* photoreceptors; potentiation of the membrane coenzyme Q reductase antioxidant pathway by overexpression of its rate-limiting partner may offer a targeted antioxidant gene therapy to counteract lipid peroxidation-induced damage.

Calcium overload induces oxidative stress, a pathophysiology associated with several diseases including excitotoxicity and reperfusion injury.^38^ The calcium-based phototransduction pathway in *Drosophila* has been used to examine the effect of calcium overload on photoreceptor health.^20^ Calcium overload in photoreceptors expressing constitutively open Trp calcium channels results in oxidative stress and retinal degeneration,^15,39^ which can be suppressed by overexpressing proteins that reduce cytosolic calcium levels.^16^ The Trp-dependence of acute photodamage observed in our study is consistent with mitochondrial ROS as a proximal initiator of photoreceptor lipid peroxidation; we speculate Cyt-b5 enhances antioxidant chain-breaking to limit further damage. Parallel to our observations, expression of mouse glutathione peroxidase (mGpx1) in fly photoreceptors retards rhabdomere loss in a Huntington’s disease/oxidative stress model.^40^ Like Cyt-b5, mGpx1 draws reducing power from cell metabolism and was the most neuroprotective of the antioxidants tested.^40^ Together, Gpx1 and Cyt-b5 may provide a powerful synergistic approach to counter lipid peroxidation in neurons exposed to strong oxidative stress during neurodegenerative disease.

Surprisingly, our data indicate that 1-day-old flies are resistant to the same level of blue light stress that induces retinal degeneration in flies <1 week later. These flies are unlikely to have aged significantly since *w* ^*1118*^ flies show peak reproductive capacity between 3 and 6 days post-eclosion at comparable temperatures (23–26 °C) to those used in our study.^41^ Despite this, it is clear that flies lacking visual pigment are more susceptible to light-induced visual senescence than pigmented flies because continuous illumination induces loss of ERG in white-eyed flies at much younger ages as compared to red-eyed strains.^42^ In these studies, continuous light-induced complete blindness in white-eyed flies by 5 days post-eclosion at 25 °C.^42^ Loss of the red visual pigment maximizes absorption of blue light,^43^ thereby increasing susceptibility to blue light-induced retinal degeneration.^11^ Our data suggest that normal light exposure (12:12 light/dark) induces oxidative stress in fairly young white-eyed flies because *w* ^*1118*^ day 6 flies show higher levels of H_2_O_2_ than their day 1 counterparts even before being exposed to blue light (Fig. 6a). This oxidative stress does not include substantial lipid peroxidation because we do not observe a similar increase in MDA levels in day 6 flies in the absence of blue light (Fig. 6b). Flies lacking visual pigment might therefore be predisposed to premature visual senescence and enhanced susceptibility to light stress.

Why then, are newly eclosed flies resistant to blue light stress? One possibility is that very young flies have lower basal levels of oxidative stress and can therefore withstand the additional blue light exposure. An alternative explanation is that newly eclosed flies are transitioning from development into early adulthood and have additional cellular mechanisms available to deal with exogenous stress. Our data do not currently distinguish between these models.

Membrane-associated and the rate-limiting electron donor for Cyt-b5 reductase,^28^ Cyt-b5 is strategically positioned to quench toxic lipid peroxidation at its source. Enhanced regeneration of coenzyme Q, which in turn recycles vitamins C and E^2,37^ suggests Cyt-b5 could synergize with antioxidant dietary supplementation of membrane antioxidants. Our data indicate that increasing Cyt-b5 levels in *Drosophila* eyes enhances the enzymatic, renewable antioxidant activity of the cell itself, and suggests a strategy to improve photoreceptor health.

## Methods

### Stocks and genetics

Flies were cultured on standard cornmeal food at 23 °C–25 °C. The genotypes used in this study are described in Supplementary Table 1 and the corresponding genes are described in Supplementary Table 2. Transgenic lines with the mini-white marker were crossed to *cn bw* to deplete eye pigments.^44^ Flies were maintained with 12 h/12 h light/dark cycle except for *ninaE* ^*7*^ and *trp* ^*9*^ flies, which together with the *w* ^*1118*^ controls for those experiments, were raised in the dark prior to blue light treatment to prevent light-dependent retinal degeneration.^45^ Mated male flies were used for all experiments unless otherwise stated. For ageing experiments, flies were collected 0–8 h post-eclosion and aged post-collection for 12 h (day 1; 12–19 h), 3 days (day 1 + 2 days), or 6 days (day 1 + 5 days).

### Blue light treatment

Flies were exposed to 2–8 h of blue light (*λ* = 465 nm) at 8000 lux (2 mW/cm^2^) using a custom designed optical stimulator with temperature control (23–25 °C). The design and characterization of the optical stimulator are fully described in ref. ^12^. Briefly, flies were placed in transparent polystyrene 25 × 99 mm vials (VWR, #89092-722), exposed to defined intensities of blue light, and light intensity was measured and exposure time was controlled using the device software. Generally, 5–10 male flies of the indicated age and genotype were exposed to blue light for indicated period of time, and compared to flies incubated in the dark for the same period of time (dark control). To assess retinal degeneration, flies were exposed to 8 h blue light or dark control, then placed in dark for 12 days to allow time for rhabdomeres to degenerate. Rhabdomere loss was assessed using immunostaining and confocal microscopy. For electron microscopy, flies were examined immediately following 8 h blue light exposure, or placed in the dark for 12 days following blue light exposure and then examined. To assay H_2_O_2_ levels and lipid peroxidation prior to retinal degeneration, eyes were dissected and assayed immediately following 2 h of blue light exposure, which is not sufficient to induce retinal degeneration in day 6 flies (data not shown).

### Immunostaining and confocal microscopy

Rhabdomere loss was assessed by immunostaining dissected retinas with phalloidin and anti-Rhodopsin 1. Briefly, eyes were dissected and fixed in phosphate-buffered saline (1XPBS, pH 7.4) containing 4% paraformaldehyde, 1 mM EGTA, 1 mM MgCl_2_ for 25 min, washed four times in PBST (PBS containing 0.3% (v/v) Triton X-100), and incubated with mouse anti-Rh1 (1:50, Developmental Studies Hybridoma Bank, Cat# 4C5) and phalloidin (1:100, ThermoFisher Scientific, Cat# A22287) for 8–16 h at 4 °C. Eyes were washed four times in PBST, incubated with secondary antibody (ThermoFisher Scientific, Cat# A21202) for 8–16 h at 4 °C, washed four times in PBST, and mounted in 2×PBS containing 70% sorbitol (w/v) (Sigma, Cat# S1876). Images were captured using a Zeiss LSM710 confocal microscope with Plan-Apochromat 63×/1.4 oil objective at Z-stack step size of 1 μm. Rhabdomere loss was quantified using stacked images and is presented as percentage loss of R1–R6 cells per ommatidium for >50 ommatidia per imaged retina. Rhabdomere loss was quantified in five independent male flies (single eye/fly) for four independent light exposures (paired blue light vs. dark controls).

### Electron microscopy

Retinas of adult flies were microinjected with pre-fixative solution (2% paraformaldehyde, 2% glutaraldehyde, 0.1 M cacodylate buffer pH 7.4) and dissected after 10 min. Fixed eyes were then incubated for more than 2 h at 4 °C. Eyes were washed five times in 0.1 M cacodylate buffer (pH 7.4), post-fixed in 2% OsO_4_ in 0.1 M cacodylate buffer for >2 h, and stained with 2% uranyl acetate for 8–16 h. Next, eyes were washed three times in deionized H_2_O, followed by dehydration with a series of ethanol washes (30% for 10 min, 50% for 10 min, 70% for 10 min, 90% for 10 min, two times in 100% for 10 min). Eyes were then incubated two times in xylene for 15 min each, transferred to 50% xylene: 50% epon (v/v) resin for 12 h, then incubated in 100% epon for 8–16 h, and finally embedded in Quetol-812 (EMS). Ultrathin sections (80 nm) were stained for 3 min with lead hydroxide. Images were collected with a Philips 300 electron microscope. The membrane fraction in the cytoplasm was quantified by quantifying the area containing membrane structures (excluding mitochondria or lysosomes) and dividing this area by the total area in the photoreceptor. For blue light aging experiments, we quantified at least one eye for five independent male flies (single eye/fly) for four independent experiments (blue light vs. dark control). For transgene overexpression experiments, three to four representative eyes from three independent male flies (single eye/fly) were quantified. For all eyes, multiple planes were analyzed across many different ommatidia to obtain estimates of membrane structures within R1–R6 cells.

### RNA isolation and qPCR analysis

RNA was isolated from dissected eyes using Trizol (Invitrogen) and quantitative PCR (qPCR) analysis was performed on cDNA generated from 100 ng RNA using oligo-dT primers relative to a standard curve of serially diluted cDNA. Relative expression for *cytochrome-b5* was normalized to the geometric mean of two reference genes (*eIF1a* and *RpL32*). Primers are listed in Supplementary Table 3.

### H_2_O_2_ assays in dissected eyes

The Amplex Red Hydrogen Peroxide/Peroxidase Assay Kit (ThermoFisher Scientific, Cat# A22188) was used to quantify the concentration of H_2_O_2_ in fly eyes based on the manufacturer’s instructions. Ten eyes per sample were dissected in ice-cold PBS (1× reaction buffer), transferred to 80 µL of fresh PBS with protease inhibitor cocktail (cOmplete protease inhibitor cocktail, Roche Diagnostics) and homogenized using a mechanized Kontes pellet pestle grinder (Kimble Chase, VWR Cat# KT749520-0000). The homogenized sample was centrifuged at 5000 x *g* for 30 s to remove cellular debris, and 50 µL of the supernatant was added to 50 µL of the Amplex Red reagent/HRP working solution in a 96-well microplate well. The standard curve was generated using 2 µM, 1 µM, 0.5 µM, and 0 µM of H_2_O_2_ standards. Fluorescence was measured with an EnVision Multilabel Plate Reader (APerkinElmer, Cat# 2104-0010) at 590 ± 20 nm for three technical replicates per sample. All experiments were performed within 30 min at room temperature. The protein concentration of the extract was determined using Qubit Protein Assay Kit (ThermoFisher, Cat# Q33211). H_2_O_2_ levels were determined as µM H_2_O_2_ /µg protein. Five biological replicates (independent light exposure, paired blue light vs. dark controls) were quantified per sample.

### Lipid peroxidation assays

The Lipid Peroxidation (MDA) Assay Kit (Colorimetric/Fluorometric) (Abcam, Cat# ab118970) was used as per the manufacturer’s instructions. Ten eyes per sample were dissected in cold 1×PBS, and then transferred to 50 µL ddH_2_O with 1 µL BHT. After homogenization, 50 µL 2 N perchloric acid was added to the slurry. Samples were vortexed and centrifuged to remove precipitated protein. 100 µL of the supernatant was added to 300 µL thiobarbituric acid reagent, and incubated at 95 °C for 1 h. 200 µL of standard or sample were added to individual wells of a GREINER 96 F-BOTTOM plate. For fluorometric measurement, signals were collected with a CLARIOStar reader (BMG LABTECH GmbH) (Ex/Em = 532 ± 8/553 ± 8 nm). Three biological replicates (independent light exposure, paired blue light vs. dark controls) were quantified per sample.

### Fatty acid analysis

The fatty acid composition of *Drosophila* eyes was analyzed by acidic transmethylation according to ref. 46 with minor modifications. Ten dissected eyes per sample were placed in glass tubes containing 1 mL 5% H_2_SO_4_ in methanol, with 300 μL of toluene as a co-solvent, 40 µg of butylated hydroxytoluene in methanol as an antioxidant, and 5 or 10 μg of tripentadecanoin (Tri-15:0-triacylglycerol) as an internal standard. Samples were heated for 1.5 h at 85–90 °C. After cooling to room temperature, the resulting fatty acid methyl esters, (FAMEs) were extracted by adding 1.5 mL of 0.9% NaCl and 1 mL of hexane. The hexane (top layer) was removed into a new glass tube, completely dried with N_2_, and then dissolved in 50 μL of hexane. FAMEs were detected by gas chromatography (GC) using flame ionization detection (Agilent 7890 A) and a FAMEWAX capillary column (30 m length × 0.25 mm i.d., 0.25 µm film thickness; Restek Cat # 12497) with helium as the carrier gas at 1.5 mL/min constant flow. For each GC run, 1 μL of sample was injected in split mode using a 10:1 ratio with the inlet set to 260 °C. The oven temperature was held at 150 °C for 3 min after injection, then increased at 10 °C/min to 240 °C, and held for 5 min. Fatty acid species in the samples were identified by comparing their retention times with FAME standards. Three biological replicates were quantified per sample.

### Electroretinogram

Male flies were attached on their side to a coverslip using nail polish and oriented with the upward eye facing red (625–630 nm) and blue (465–470 nm) superbright LEDs driven by a Grass S88 stimulator. *Drosophila* Ringer-filled pulled glass pipettes were inserted into the retina and into the thorax for recording using a Getting Model 5 microelectrode amplifier and AD Instruments Powerlab and Lab Chart. Flies were dark adapted and then exposed to the blue LED for 4 s, then again 20 and 40 s later. A red flash was used to photorevert metarhodopsin to rhodopsin before the next recording. Representative traces are shown.

### Vitamin E and lipoic acid supplementation

Newly eclosed male *w* ^*1118*^ flies were cultured on standard cornmeal food supplemented with vitamin E or lipoic acid for 6 days prior to blue light exposure. 200 μM vitamin E (Sigma, Cat# 258024) or 2.15 mM lipoic acid (Sigma, Cat# T1395) dissolved in 100% ethanol was added to the top of standard cornmeal food in a vial, mixed, and left overnight to allow the ethanol to evaporate. An equivalent volume of ethanol was added to standard fly food as control (vehicle).

### Statistics

Data were plotted and statistical tests were performed using built-in R functions (v3.4.1) and custom scripts, which are available upon request. Sample sizes were not pre-determined. Flies were assigned randomly to control or treatment groups without blinding. Box plots with overlayed points were generated using ggplot2; lower and upper hinges correspond to the first and third quartiles, and the whiskers extend to the smallest or largest values no more than 1.5× inter-quartile range from each hinge. For experiments with two groups of data, unpaired Student’s *t* tests were performed. For experiments with more than two groups, data were first tested for normal distribution. Normally distributed data were analyzed using ANOVA followed by Tukey’s honest significant different (HSD) post hoc test. If data were not normally distributed, we performed Kruskal–Wallis tests followed by pairwise Wilcoxon Rank Sum Tests and determined FDR values using a Benjamini and Hochberg correction.

### Data availability

All raw and supporting data has been deposited at the Purdue University Research Repository (PURR) as a publically available, archived data set and can be accessed using https://doi.org/10.4231/R789141Q. Any additional scripts or material required for analysis are available from the corresponding author on reasonable request.

## Electronic supplementary material

Supplementary Material

## Electronic supplementary material

**Supplementary information** accompanies the paper on the *npj Aging and Mechanisms of Disease* website (10.1038/s41514-017-0019-6).
