# Supplementary material for: Cytochrome b5 protects photoreceptors from light stress-induced lipid peroxidation and retinal degeneration
Source: NPJ Aging Mech Dis. 2017 Dec 4;3:18. doi: 10.1038/s41514-017-0019-6 (PMC5712525; doi:10.1038/s41514-017-0019-6)
Supplement: Supplementary file 1 — Supplementary Material [file 41514_2017_19_MOESM1_ESM.pdf]

SUPPLEMENTARY MATERIAL

Chen *et al.* Cytochrome b5 protects photoreceptors from light stress-induced lipid peroxidation and retinal degeneration

LIST OF SUPPLEMENTARY MATERIAL

TABLE S1: Fly stocks used in this study

TABLE S2: Primers used in this study.

TABLE S3: Primers used in this study.

SUPPLEMENTARY FIGURE LEGENDS

FIGURE S1: Cyt-b5 transcript levels are not reduced in day 6 flies.

FIGURE S2: The Cyt-b5 transgene does not suppress blue light-induced retinal degeneration due to position (insertion) effect.

FIGURE S3: Fatty acid composition in the eye is not altered by overexpression of Cyt-b5.

FIGURE S4: Blue light-induced increase in H_2O_2 levels requires Ca^{2+} influx.

FIGURE S5: Overexpression of Cyt-b5 does not inhibit visual function.

FIGURE S6: Treatment with Vitamin E, but not overexpression of Sod1, partially suppresses blue light-induced retinal degeneration.

Fly stock	Detailed genotype	Source	Stock number	Reference
w^{1118}	w^{1118}	Bloomington	BL3605	[1]
$ninaE^{7}$	w^{1118};; $ninaE^{7}$	Bloomington	BL1613	[2,3]
trp^{9}	w^{*};; trp^{9}	Bloomington	BL9046	[4]
LGMR-Gal4	y^{1} w^{*}; wg^{Sp-1}/CyO; P{longGMR-GAL4}3/TM2	Bloomington	BL8121	[5]
UAS-luciferase	w^{*}; P{nSyb-MKII::GAL4DBDo}attP24, P{QUAS-p65AD::CaM}2/CyO; P{UAS-LUC.D}3	Bloomington	BL61678	[6]
UAS-Cyt-b5	*P{UAS-dappled}3b*	Deborah Kimbrell		[7]
UAS-Hmgcr	*P{UAS-Hmgcr.vD}3*	Ruth Lehmann		[8]
UAS-Sod1	w^{1};; P{UAS-Sod1.A}B37	Bloomington	BL24750	[9]
cn bw	cn^{1} bw^{1}	Bloomington	BL264	[10]
$cytb5^{01857}$	P{PZ}$Cyt\text{-}b5^{01857}$, cn^{1}/CyO; ry^{506}	Bloomington	BL11101	[7,11]
cn bw; LGMR-Gal4	cn^{1} bw^{1}; P{longGMR-GAL4}e3/TM2	This study		
cn bw; UAS-luciferase	cn^{1} bw^{1}; P{UAS-LUC.D}3	This study		
cn bw; UAS-Cyt-b5	cn^{1} bw^{1}; P{UAS-dappled}3b	This study		
cn bw; UAS-Hmgcr	cn^{1} bw^{1}; P{UAS-Hmgcr.vD}	This study		
cn bw, $cytb5^{01857}$	cn^{1} bw^{1} $cytb5^{01857}$/CyO	This study		
cn bw; UAS-Sod1	*cn bw; P{UAS-Sod1.A}B37*	This study		

Table S1. Fly stocks used in this study.

SUPPLEMENTARY REFERENCES FOR TABLE S1

1 Rabinow, L. & Birchler, J. A. A dosage-sensitive modifier of retrotransposon-induced alleles of the Drosophila white locus. *EMBO J* **8**, 879-889 (1989).

2 Kumar, J. P. & Ready, D. F. Rhodopsin plays an essential structural role in Drosophila photoreceptor development. *Development* **121**, 4359-4370 (1995).

3 Kurada, P. & O'Tousa, J. E. Retinal degeneration caused by dominant rhodopsin mutations in Drosophila. *Neuron* **14**, 571-579 (1995).

4 Wang, T., Jiao, Y. & Montell, C. Dissecting independent channel and scaffolding roles of the Drosophila transient receptor potential channel. *J Cell Biol* **171**, 685-694, doi:10.1083/jcb.200508030 (2005).

5 Wernet, M. F. *et al.* Homothorax switches function of Drosophila photoreceptors from color to polarized light sensors. *Cell* **115**, 267-279 (2003).

6 Gao, X. J. *et al.* A transcriptional reporter of intracellular Ca(2+) in Drosophila. *Nat Neurosci* **18**, 917-925, doi:10.1038/nn.4016 (2015).

7 Kleinhesselink, K., Conway, C., Sholer, D., Huang, I. & Kimbrell, D. A. Regulation of hemocytes in Drosophila requires dappled cytochrome b5. *Biochem Genet* **49**, 329-351, doi:10.1007/s10528-010-9411-7 (2011).

8 Van Doren, M., Broihier, H. T., Moore, L. A. & Lehmann, R. HMG-CoA reductase guides migrating primordial germ cells. *Nature* **396**, 466-469, doi:10.1038/24871 (1998).

9 Anderson, P. R., Kirby, K., Hilliker, A. J. & Phillips, J. P. RNAi-mediated suppression of the mitochondrial iron chaperone, frataxin, in Drosophila. *Hum Mol Genet* **14**, 3397-3405, doi:10.1093/hmg/ddi367 (2005).

10 Tearle, R. Tissue specific effects of ommochrome pathway mutations in Drosophila melanogaster. *Genet Res* **57**, 257-266 (1991).

11 Russell, M. A., Ostafichuk, L. & Scanga, S. Lethal P-lacZ insertion lines expressed during pattern respecification in the imaginal discs of Drosophila. *Genome* **41**, 7-13 (1998).

Gene	Symbol	Flybase ID	Mutant allele
white	w	FBgn0003996	w^{1118}
neither inactivation nor afterpotential E	*ninaE (Rh1)*	FBgn0002940	$ninaE^{7}$
transient receptor potential	*trp*	FBgn0003861	trp^{9}
Cytochrome b5	*Cyt-b5*	FBgn0264294	$cytb5^{01857}$
HMG Coenzyme A reductase	*Hmgcr*	FBgn0263782	
Superoxide dismutase 1	*Sod1*	FBgn0003462	
cinnabar	*cn*	FBgn0000337	cn^{1}
brown	*bw*	FBgn0000241	bw^{1}

Table S2. Genes examined in this study.

**Gene**	**Symbol**	**Flybase ID**	**Primer (5' – 3')**
Cytochrome b5	*Cyt-b5*	FBgn0264294	F: CCAAGCACAACACGAACAAG<br>R: GCCAACGTCCTCAAAGTTCT
eukaryotic translation initiation factor 1A	*eIF1A*	FBgn0026250	F: GCTGGGCAACGGTCGTCTGGAGGC<br>R: CGTCTTCAGGTTCCTGGCCTCGTCCGG
Ribosomal protein L32	*RpL32*	FBgn0002626	F: GCTAAGCTGTCGCACAAATG<br>R: CGTTGTGCACCAGGAACTT
Luciferase	*Luc*		F: CAACTGCATAAGGCTATGAAGAGA<br>R: ATTTGTATTCAGCCCATATCGTTT

Table S3. Primers used in this study.

SUPPLEMENTARY FIGURE LEGENDS

Fig. S1. *Cyt-b5* transcript levels are not reduced in day 6 flies. Bar plot showing qPCR analysis of *Cyt-b5* in dissected eyes from male flies of the indicated ages and genotype. The expression of *Cyt-b5* was normalized to the geometric mean of two reference genes (*eIF1A* and *RpL32*) and is shown relative to day 1 w^{1118}, which is set to one. Data represent mean ± s.d. (n = 3). *p* values, Student's t-test between indicated samples. ns, not significant.

Fig. S2. The *Cyt-b5* transgene does not suppress blue light-induced retinal degeneration due to position (insertion) effect. Confocal images of adult retinas from flies carrying single copy transgenes for *LGMR-Gal4, Luciferase* or *Cyt-b5* in the *cn bw* background exposed to 8h blue light at 6 days post-eclosion. Scale bars: 10 μm. Mean percentage rhabdomere loss is shown (*n = 2 light treatments; 5 animals/experiment*).

Fig. S3. Fatty acid composition in the eye is not altered by overexpression of Cyt-b5. (A) Retinas were dissected from adult male w^{1118} flies 1 and 6 days post-eclosion, or 6 day old male flies expressing single copy of the indicated *UAS* transgenes driven by *LGMR-Gal4* in the *cn bw* background. Fatty acids were extracted and quantified. The molar percentage of each fatty acid was determined for each sample. (B) The ratio of 16:1 to 16:0 fatty acids were determined for each sample. (C) The ratio of unsaturated to saturated fatty acids were compared for each sample. All data represent mean ± s.d. (*n = 3 for all samples except UAS-luciferase with n = 2*). *p*-values indicated by asterisks for significant comparisons were determined by ANOVA followed by Tukey-HSD using GraphPad Prism 7.03 between w^{1118} day 6 and indicated age/genotype (*, $p < 0.05$; ** $p < 0.01$). *UAS-luciferase* samples were not included in the statistical analysis.

Fig. S4. Blue light-induced increase in H_2O_2 levels requires Ca^{2+} influx. H_2O_2 levels were measured in retinas from w^{1118} and trp^9 flies exposed to 2h blue light or dark control at 6 days

post-eclosion. Flies were raised in the dark prior to blue light exposure. H_2O_2 concentrations were determined relative to total amount of protein from 10 eyes, and data are plotted as individual points. FDR, pairwise Wilcoxon Rank Sum Test between different genotypes under the same light treatment (*n = 5 light treatments*).

Fig. S5. Overexpression of Cyt-b5 does not inhibit visual function. Electroretinograms (ERG) performed on 6 day old flies expressing single copy transgenes for *Luciferase* or *Cyt-b5* driven by *LGMR-Gal4* in the *cn bw* background. Light pulses (4s pulse with 20s intervals, red or blue light) are indicated by the bars, and ERG recordings are shown by the red trace.

Fig. S6. Treatment with Vitamin E, but not overexpression of Sod1, partially suppresses blue light-induced retinal degeneration. (A) Confocal images of adult retinas from flies expressing single copy transgenes for *Sod1* or *Luciferase* driven by *LGMR-Gal4* in the *cn bw* background exposed to 8h blue light or dark at 6 days post-eclosion. (B) Confocal images of adult retinas from w^{1118} flies grown on food supplemented with Vitamin E (VitE) or vehicle control (ethanol) for 6 days post-eclosion prior to exposure to 8h blue light or dark control. (C) Box plots showing rhabdomere loss quantified using the confocal images. FDR, pairwise Wilcoxon Rank Sum Test between genotypes or Vitamin E treatment under the same light exposure (*n = 4 light treatments; 5 animals/experiment*). ns, not significant. (D) H_2O_2 levels were measured in retinas from flies of the indicated genotypes or Vitamin E treatment exposed to 2h blue light or dark control. H_2O_2 concentrations were determined relative to total amount of protein from 10 eyes, and data are plotted as individual points. *p*-values, ANOVA followed by Tukey-HSD between different genotypes or treatment under the same light exposure (*n = 5 light treatments*). ns, not significant.

Chen_FigS1

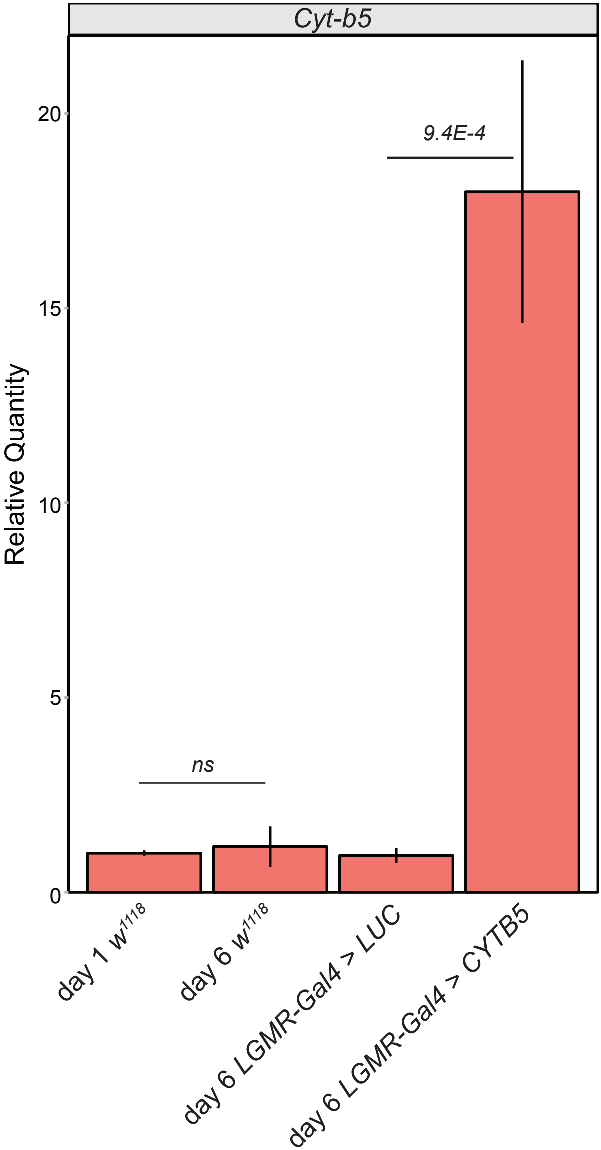

Cyt-b5
Relative Quantity
0
5
10
15
20
9.4E-4
ns
day 1 w1118
day 6 w1118
day 6 LGMR-Gal4 > LUC
day 6 LGMR-Gal4 > CYTB5

Chen_FigS2

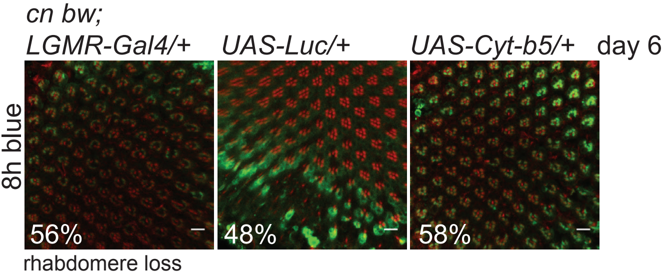

cn bw;
LGMR-Gal4/+
UAS-Luc/+
UAS-Cyt-b5/+
day 6
8h blue
56%
48%
58%
rhabdomere loss

Chen_FigS3

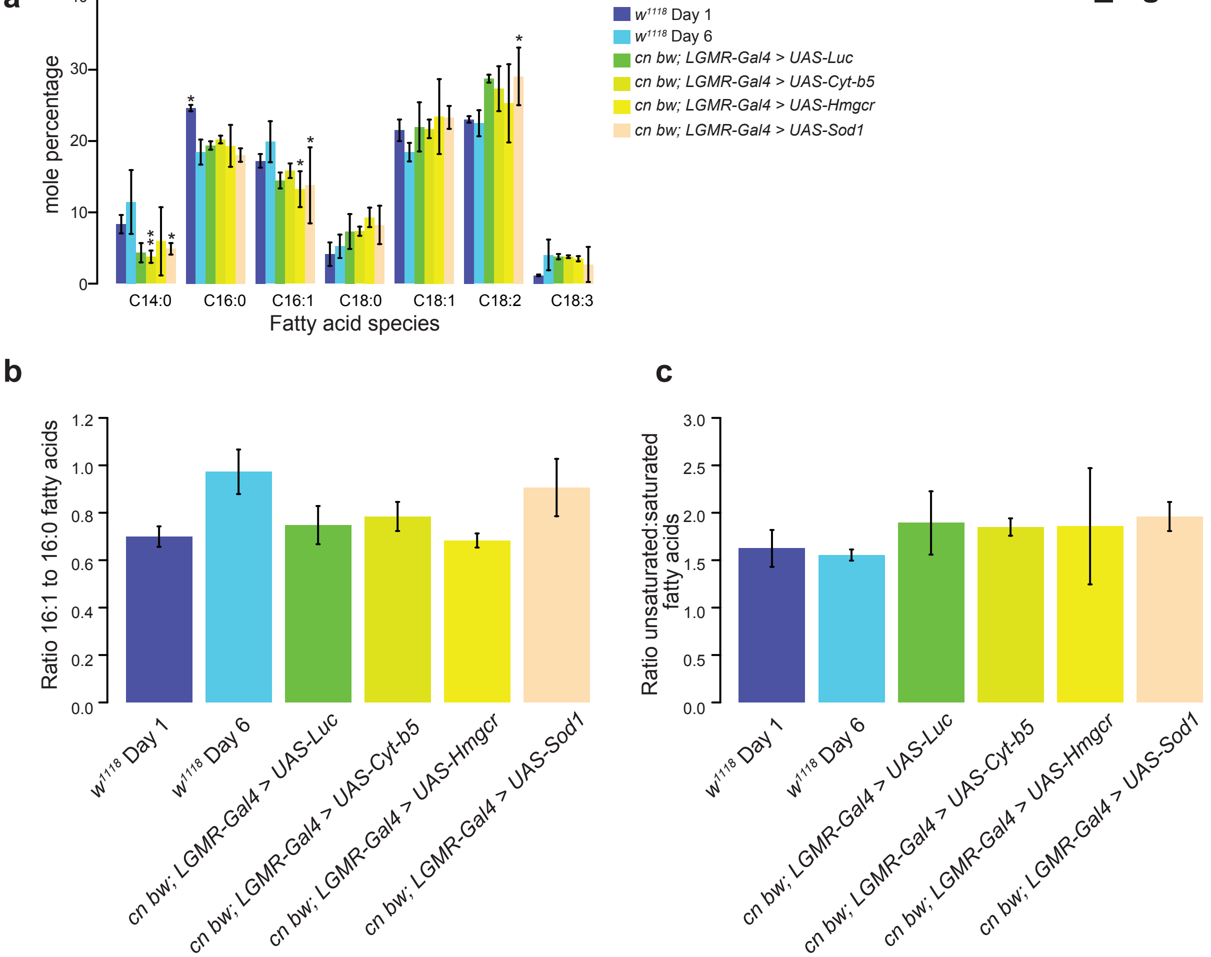
a
mole percentage
40
30
20
10
0
C14:0
C16:0
C16:1
C18:0
C18:1
C18:2
C18:3
Fatty acid species
*
w1118 Day 1
w1118 Day 6
cn bw; LGMR-Gal4 > UAS-Luc
cn bw; LGMR-Gal4 > UAS-Cyt-b5
cn bw; LGMR-Gal4 > UAS-Hmgcr
cn bw; LGMR-Gal4 > UAS-Sod1
b
Ratio 16:1 to 16:0 fatty acids
1.2
1.0
0.8
0.6
0.4
0.2
0.0
w1118 Day 1
w1118 Day 6
cn bw; LGMR-Gal4 > UAS-Luc
cn bw; LGMR-Gal4 > UAS-Cyt-b5
cn bw; LGMR-Gal4 > UAS-Hmgcr
cn bw; LGMR-Gal4 > UAS-Sod1
c
Ratio unsaturated:saturated fatty acids
3.0
2.5
2.0
1.5
1.0
0.5
0.0
w1118 Day 1
w1118 Day 6
cn bw; LGMR-Gal4 > UAS-Luc
cn bw; LGMR-Gal4 > UAS-Cyt-b5
cn bw; LGMR-Gal4 > UAS-Hmgcr
cn bw; LGMR-Gal4 > UAS-Sod1

Chen_FigS4

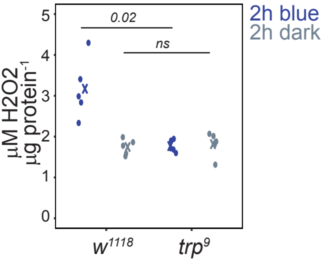

Chen_FigS5

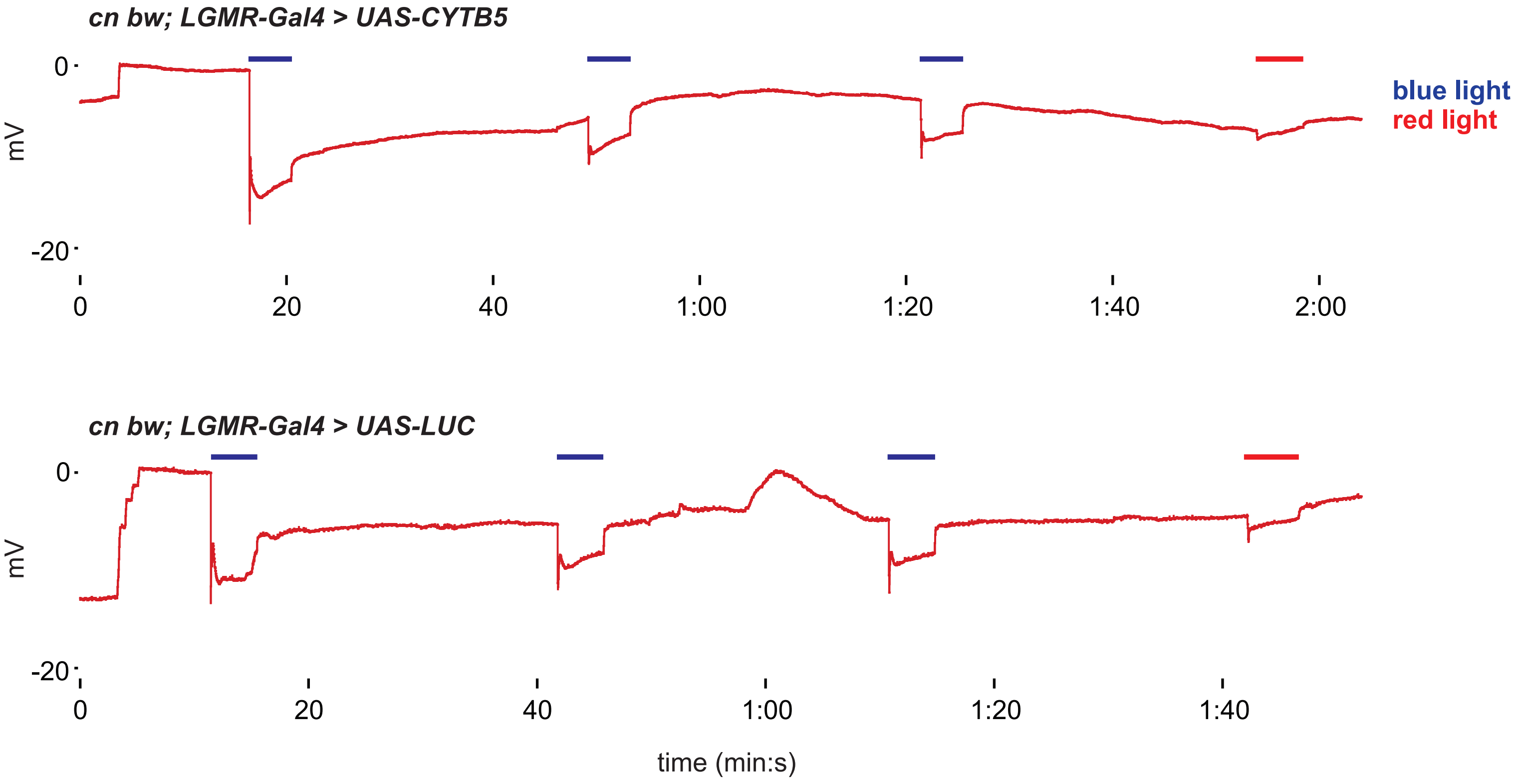

cn bw; LGMR-Gal4 > UAS-CYTB5
blue light
red light
0
-20
mV
0
20
40
1:00
1:20
1:40
2:00
cn bw; LGMR-Gal4 > UAS-LUC
0
-20
mV
0
20
40
1:00
1:20
1:40
time (min:s)

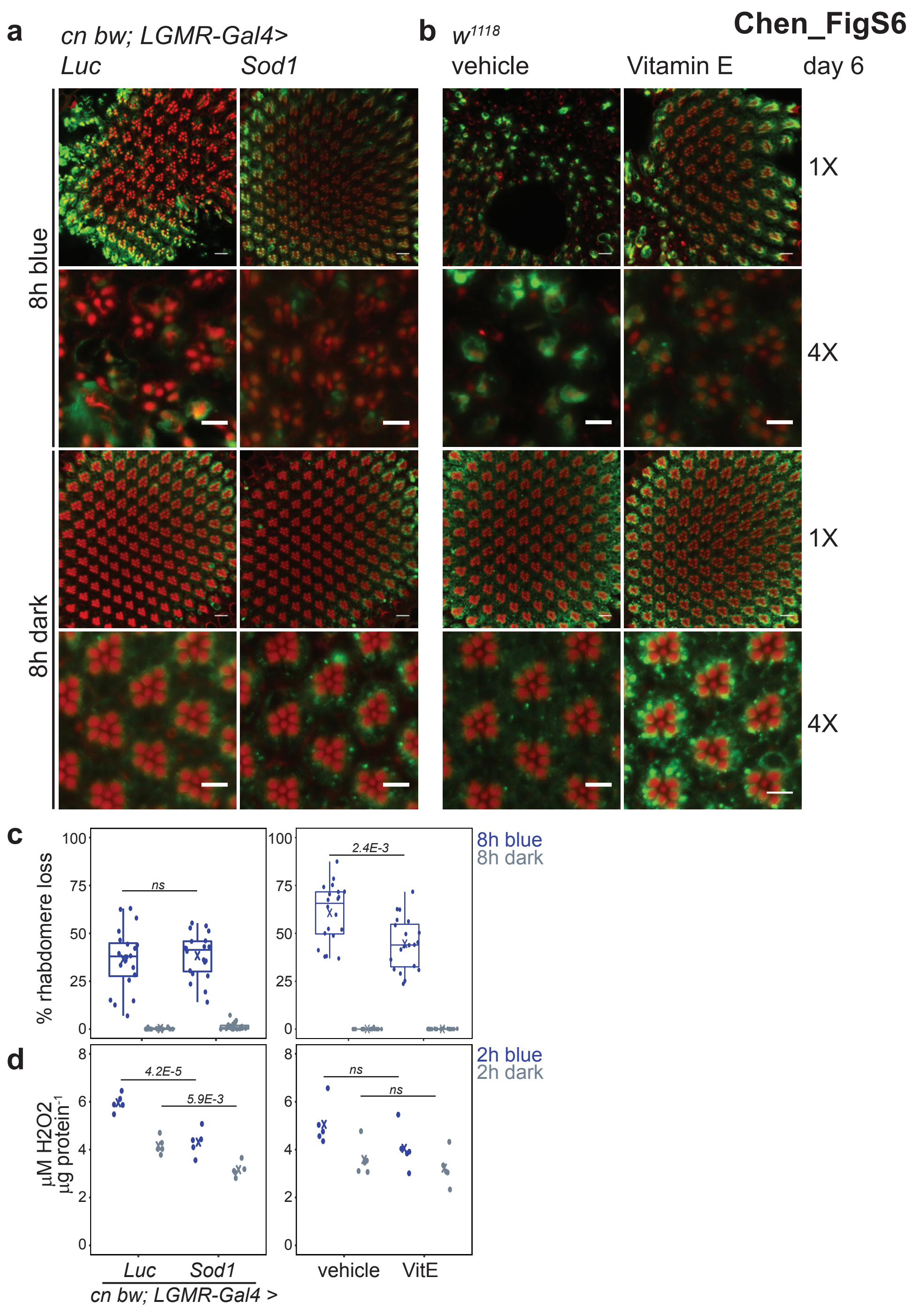
Chen_FigS6
a
cn bw; LGMR-Gal4>
Luc
Sod1
b
w1118
vehicle
Vitamin E
day 6
1X
4X
8h blue
8h dark
c
% rhabdomere loss
ns
2.4E-3
8h blue
8h dark
d
μM H2O2 μg protein-1
4.2E-5
5.9E-3
ns
2h blue
2h dark
Luc
Sod1
cn bw; LGMR-Gal4 >
vehicle
VitE
